# Pathophysiological Variants of Horizontal Semicircular Canal Benign Paroxysmal Positional Vertigo: Toward an Integrative Model Based on Otoconial Location and Cupular Dynamics

**DOI:** 10.3390/audiolres16040117

**Published:** 2026-08-13

**Authors:** Joan Lorente-Piera, Raquel Manrique-Huarte, Nicolás Pérez-Fernández

**Affiliations:** 1 Department of Otorhinolaryngology, Complejo Hospitalario Universitario Insular Materno-Infantil, 35016 Las Palmas de Gran Canaria, Spain; 2 Department of Otorhinolaryngology, Clínica Universidad de Navarra, 31008 Pamplona, Spain; 3 Department of Otorhinolaryngology, Clínica Universidad de Navarra, 28027 Madrid, Spain

**Keywords:** benign paroxysmal positional vertigo, canalolithiasis, cupulolithiasis, positional nystagmus, geotropic, apogeotropic

## Abstract

**Objective:** To critically synthesize the available evidence on the pathophysiological variants of horizontal semicircular canal benign paroxysmal positional vertigo (HSC-BPPV) and to propose an integrative model based on otoconial location and cupular dynamics to explain their clinical presentation and therapeutic implications. **Methods:** A scoping review was conducted through a search of PubMed/MEDLINE, Scopus, and the Cochrane Library for studies published between January 1996 and May 2026. Studies describing pathophysiological variants, biomechanical mechanisms, nystagmus patterns, or therapeutic maneuvers in HSC-BPPV were included. A total of 26 studies met the inclusion criteria and underwent qualitative analysis. **Results:** Six principal mechanical configurations of horizontal canal positional vertigo were identified: long-arm canalolithiasis, short-arm canalolithiasis, canal-side cupulolithiasis, utricular-side cupulolithiasis, canalith jam, and light cupula. The available evidence suggests that these variants represent different mechanical states determined by otoconial location, otoconia–cupula interaction, canal obstruction, or alterations in cupula–endolymph density. This model explains the direction, intensity, and duration of positional nystagmus, the findings observed during the supine roll test and the bow and lean test, as well as the variability in response to repositioning maneuvers. A practical classification integrating pathophysiology, clinical findings, and treatment is proposed. **Conclusions:** HSC-BPPV may be more appropriately understood as a spectrum of mechanical states in which the configuration of otoconial debris and its interaction with the cupula influence the nystagmus pattern, clinical presentation, and therapeutic response. This integrative model may improve diagnostic accuracy and guide treatment selection, although prospective validation is required.

## 1. Introduction

Benign paroxysmal positional vertigo (BPPV) is the most prevalent peripheral vestibular disorder and one of the best-studied clinical models illustrating the interaction between anatomy, fluid dynamics, and vestibular neurophysiology [1,2]. Current understanding is based on the displacement of calcium carbonate particles, known as otoconia, from the utricular macula into the semicircular canals, where they produce abnormal mechanical deflection of the cupula [3]. This model is particularly applicable to the horizontal semicircular canal (HSC), whose complex spatial orientation introduces substantially greater pathophysiological variability. In addition, the approximately 30° inclination of the canal relative to the horizontal plane generates a constant gravitational component that may modulate endolymphatic dynamics even at rest [4].

Horizontal semicircular canal BPPV accounts for a substantial proportion of BPPV cases, with an overall prevalence ranging from 10% to 31% across clinical series [5,6]. According to Schubert et al. [7], the distribution of HSC-BPPV variants is not homogeneous. The geotropic variant, classically attributed to canalolithiasis of the long arm, predominates and accounts for approximately 60–70% of HSC-BPPV cases. In contrast, the apogeotropic variant, historically associated with cupulolithiasis, represents approximately 25–35% of cases. Less frequent forms include direction-fixed nystagmus caused by canal obstruction, also referred to as canalith jam, which account for fewer than 2% of cases but are clinically important because of their potential to mimic acute vestibular disorders. Rather than being merely descriptive, this distribution reflects the relative likelihood of otoconia occupying different canal segments and adopting distinct mechanical states within the same anatomical system [8]. This conceptual shift is significant because it moves the focus from a static loading model to a dynamic framework based on the interaction between otoconia, endolymphatic flow, and canal anatomy, in which the spatial distribution of particles plays a central role.

Within this framework, accumulating evidence suggests that HSC-BPPV should be regarded as a mechanical continuum rather than a collection of discrete entities. The location of otoconia within the canal not only determines the direction of endolymphatic flow generated during positional maneuvers but also largely defines the resulting nystagmus pattern [9,10]. This behavior is governed by Ewald’s second law, according to which, in the HSC, ampullopetal endolymphatic flow elicits a stronger excitatory response than ampullofugal flow [11]. Consequently, ampullopetal stimulation of the HSC activates the vestibulo-ocular pathway through contraction of the ipsilateral lateral rectus and contralateral medial rectus muscles, generating the horizontal component of the vestibulo-ocular reflex [6]. Conversely, ampullofugal stimulation produces an inhibitory response, explaining the asymmetry in nystagmus intensity according to head position and providing the physiological basis for clinical lateralization in HSC-BPPV variants [12,13].

This integrative perspective has direct clinical implications. First, it provides a coherent framework for interpreting apparently atypical nystagmus patterns, thereby reducing reliance on diagnoses of exclusion or on unproven pathophysiological mechanisms. Second, it explains the variability in therapeutic response, which depends largely on whether the underlying mechanism involves particle mobilization, cupular positioning, or relief of canal obstruction. Finally, it highlights the need to move beyond classifications based solely on nystagmus direction toward models that integrate anatomy, system mechanics, and clinical phenomenology.

Accordingly, the aim of the present review is to propose an integrative pathophysiological model of HSC-BPPV based on otoconial location and cupular dynamics. This approach seeks not only to systematically synthesize the described variants but also to provide a conceptual framework that explains the transitions between them and their correlation with clinical presentation.

## 2. Materials and Methods

### 2.1. Study Design

This scoping review was conducted and reported in accordance with the Preferred Reporting Items for Systematic Reviews and Meta-Analyses Extension for Scoping Reviews (PRISMA-ScR). The study selection process is presented using the PRISMA 2020 flow diagram. No review protocol was prospectively registered [14].

A scoping review was considered the most appropriate methodology because the available literature comprises heterogeneous evidence, including biomechanical studies, physiological models, clinical case series, and narrative reviews, precluding quantitative synthesis or meta-analysis. The primary objective was to systematically map and critically synthesize the available evidence regarding the pathophysiological variants of horizontal semicircular canal benign paroxysmal positional vertigo (HSC-BPPV) and to develop an integrative conceptual framework based on current knowledge.

### 2.2. Research Questions

The review addressed the following research questions:

- Which pathophysiological variants of HSC-BPPV have been described?
- Which biomechanical mechanisms have been proposed to explain these variants?
- How do these mechanisms account for positional nystagmus patterns and clinical findings?
- Can these mechanisms be integrated into a unified pathophysiological model?

### 2.3. Literature Search

A comprehensive literature search was performed in PubMed/MEDLINE, Scopus, and the Cochrane Library for studies published between January 1996 and May 2026.

The search combined controlled vocabulary (MeSH terms when applicable) and free-text keywords related to HSC-BPPV and its proposed variants, including combinations of the following terms: “benign paroxysmal positional vertigo”, “BPPV”, “horizontal canal”, “lateral canal”, “horizontal semicircular canal”, “canalolithiasis”, “cupulolithiasis”, “short-arm”, “canalith jam”, “light cupula”, “geotropic”, and “apogeotropic”.

The reference lists of all included articles were also manually screened to identify additional relevant publications.

### 2.4. Eligibility Criteria

Studies were included if they

- Described pathophysiological variants of HSC-BPPV;
- Investigated biomechanical mechanisms underlying positional nystagmus;
- Reported clinical findings relevant to the interpretation of HSC-BPPV variants;
- Proposed diagnostic or mechanistic models supported by clinical or experimental observations.

Studies were excluded if they

- Exclusively addressed posterior or anterior canal BPPV without relevant information regarding HSC-BPPV;
- Evaluated therapeutic outcomes without discussing pathophysiological mechanisms;
- Were conference abstracts, editorials without analytical content, or experimental studies lacking clinical relevance.

### 2.5. Study Selection and Data Charting

After duplicate removal, studies were screened in two stages consisting of title/abstract screening followed by full-text assessment according to the predefined eligibility criteria.

**Figure 1:**
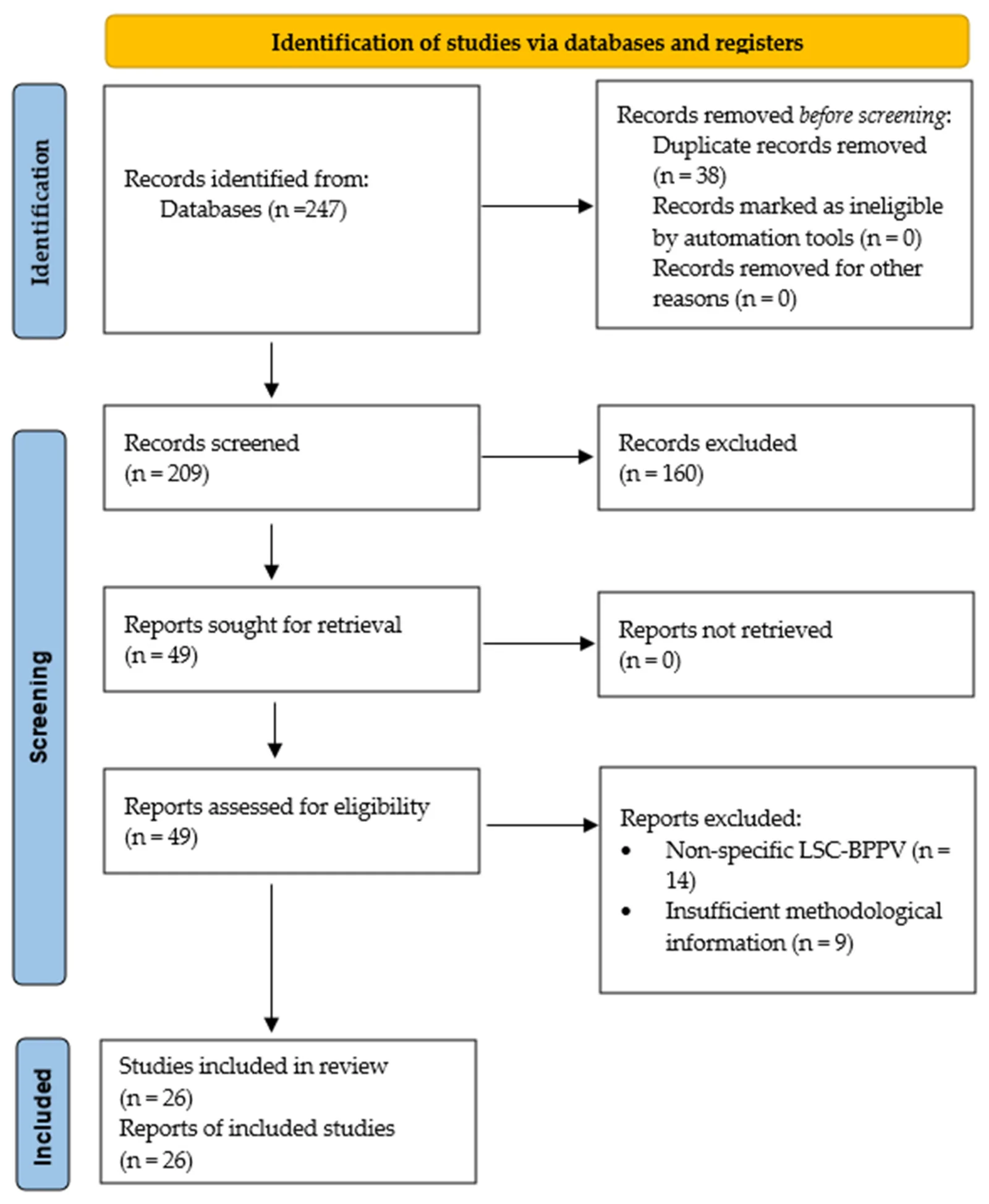
PRISMA 2020 flow diagram of the study selection process.

A total of 247 records were identified through database searching. After removal of 38 duplicate records, 209 records underwent title and abstract screening, of which 160 were excluded. Forty-nine full-text reports were assessed for eligibility, and 23 were excluded. Ultimately, 26 studies were included in the qualitative synthesis. The study selection process is summarized in the PRISMA 2020 flow diagram (Figure 1).

For each included study, information regarding study design, proposed pathophysiological mechanism, clinical presentation, positional nystagmus characteristics, diagnostic findings, therapeutic implications, and principal conclusions was extracted. No formal risk-of-bias assessment was performed because the purpose of this scoping review was to map the available evidence rather than evaluate comparative study quality.

### 2.6. Data Synthesis

Given the substantial heterogeneity of study designs, objectives, and reported outcomes, quantitative synthesis was not appropriate.

Instead, findings were synthesized descriptively to identify recurring mechanistic concepts, areas of agreement, and unresolved controversies. Based on this synthesis, an integrative pathophysiological model was developed to explain the spectrum of HSC-BPPV variants.

## 3. Results

Six principal mechanical configurations were identified from the available literature. Their main clinical characteristics, as determined by physical examination, are summarized in Table 1 and described in detail below.

### 3.1. Long-Arm (Non-Ampullary) Canalolithiasis

Long-arm HSC canalolithiasis represents the prototypical pathophysiological form of horizontal canal BPPV and is characterized by the presence of freely mobile otoconial particles within the non-ampullary segment of the canal [15]. In this setting, the otoconial aggregate acts as a generator of endolymphatic flow within the membranous labyrinth, creating a pressure gradient that is transmitted to the cupula and induces its deflection [16]. This model accounts for the latency, paroxysmal nature, and fatigability of the resulting nystagmus, while introducing an additional key concept: slow-phase velocity (SPV) is directly proportional to the mechanical energy transferred by the otoconial aggregate. Experimental models and clinical studies have consistently shown that canalolithiasis produces higher SPV values than cupulolithiasis because it represents a dynamic system capable of generating a more efficient peak of endolymphatic flow [17].

**Figure 2:**
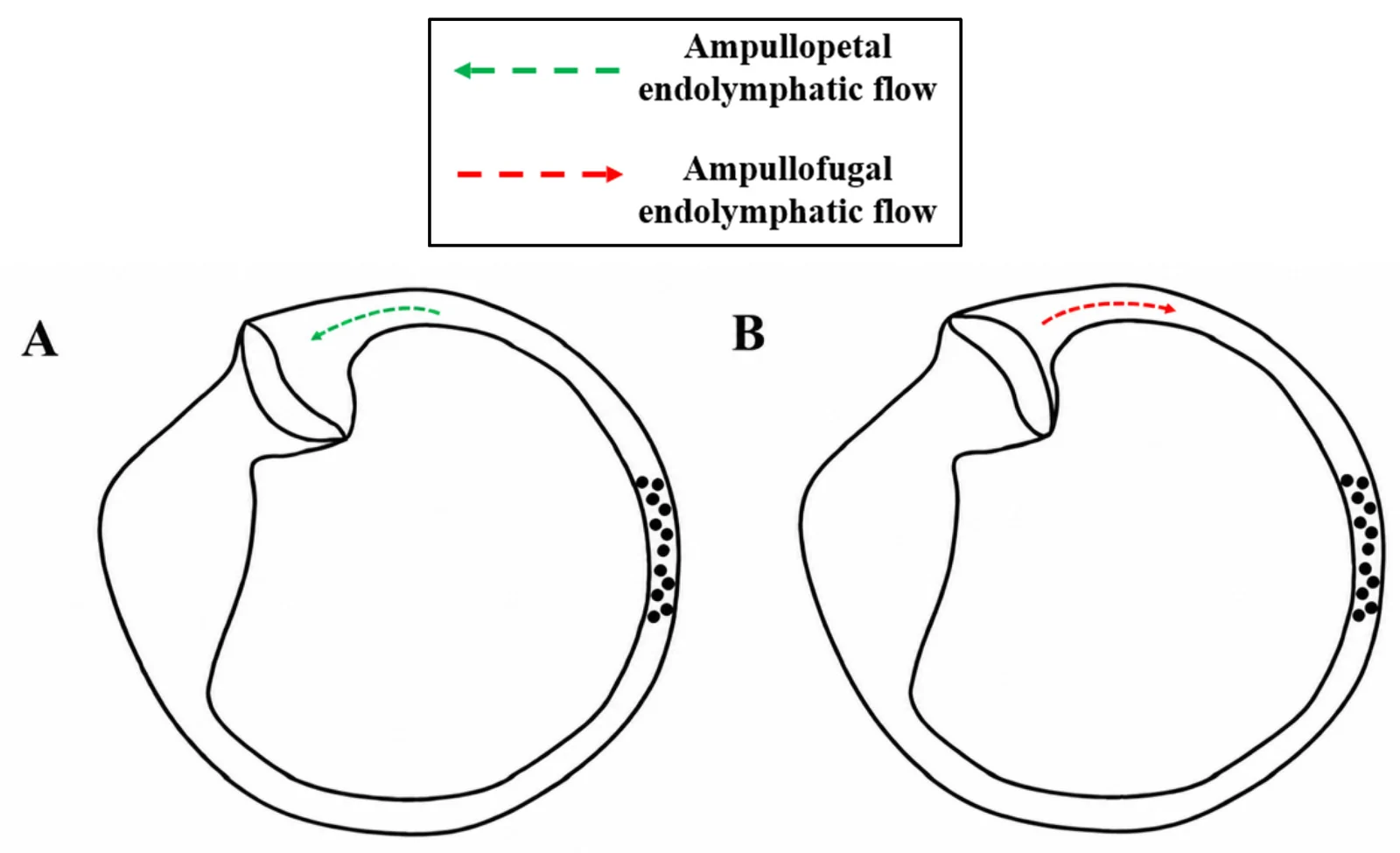
Long-arm HSC canalolithiasis (geotropic variant). (**A**) Head rotation toward the affected side: displacement of the otoconial debris toward the ampulla, generating excitatory ampullopetal endolymphatic flow. (**B**) Contralateral head rotation: displacement of the otoconial debris away from the ampulla, producing inhibitory ampullofugal endolymphatic flow.

During the supine roll test (SRT), also known as the Pagnini–McClure maneuver, performed with the patient in the supine position and the head rotated approximately 90° toward either side, rotation toward the affected ear displaces the particles toward the ampulla, generating excitatory ampullopetal endolymphatic flow and a more intense geotropic horizontal nystagmus. In contrast, rotation toward the unaffected side produces inhibitory ampullofugal endolymphatic flow of lower intensity [18]. This difference in nystagmus intensity constitutes the primary criterion for side localization [1], as illustrated in Figure 2.

The Bow and Lean test, also referred to as the Head Pitch Test, is performed in the sitting position with forward head flexion greater than 90° (Bow) and backward extension greater than 45° (Lean). It provides complementary information in the sagittal plane and may also help distinguish pseudo-spontaneous nystagmus from true spontaneous nystagmus, such as that observed in acute vestibular syndrome (AVS). Forward flexion promotes ampullopetal displacement of the otoconial aggregate, whereas head extension induces ampullofugal flow, reproducing the same vectorial pattern observed during horizontal head rotation. Nevertheless, its diagnostic yield is limited and varies across studies, being present in only approximately 30% of patients [19] and should therefore be regarded as a complementary test. Overall, the short latency, limited duration, and fatigability of the nystagmus reflect the behavior of a system dominated by transient mechanical forces and the progressive dissipation of kinetic energy, explaining both the high prevalence of this variant and its high rate of spontaneous resolution and excellent response to canalith repositioning maneuvers [18,20].

### 3.2. Short-Arm (Periampullary) Canalolithiasis

**Figure 3:**
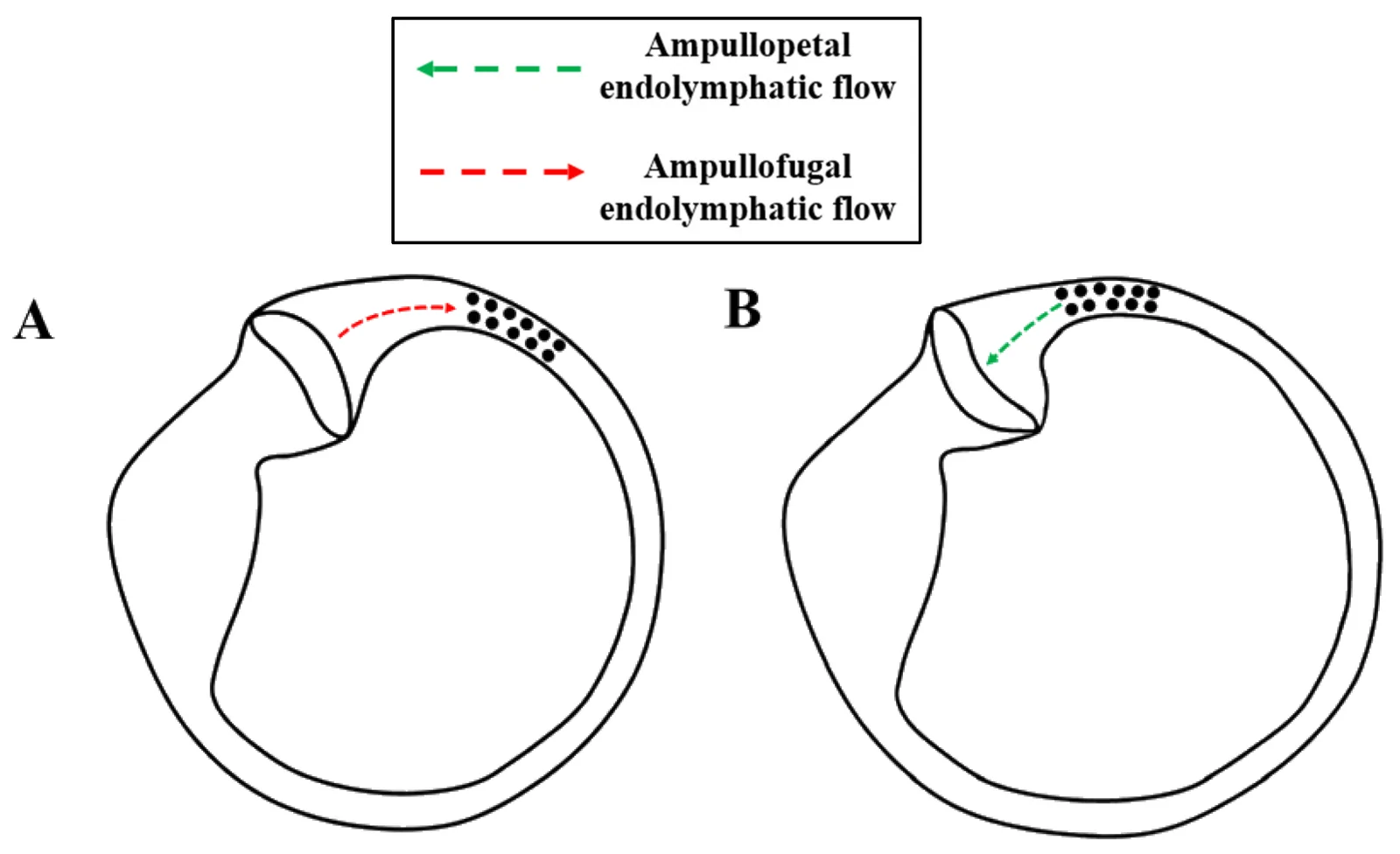
Short-arm (periampullary) HSC canalolithiasis. (**A**) Head rotation toward the affected side: displacement of the otoconial debris away from the ampulla, generating inhibitory ampullofugal endolymphatic flow and a less intense apogeotropic nystagmus. (**B**) Contralateral head rotation: displacement of the otoconial debris toward the ampulla, producing excitatory ampullopetal endolymphatic flow and a more intense nystagmic response.

Short-arm HSC canalolithiasis can be regarded as a periampullary variant of canalolithiasis, in which the otoconial debris is located within the anterior segment of the canal, as shown in Figure 3 [2]. This location creates a substantially different mechanical environment: particle displacement occurs over a short trajectory, resulting in less endolymphatic flow development and, consequently, a more limited and less sustained transfer of mechanical energy to the cupula [21]. The resulting cupular deflection is therefore governed by the complex anatomical environment adjacent to the vestibule, attenuating the classical paroxysmal pattern and producing shorter and less stereotyped responses [22].

From a pathophysiological perspective, this variant challenges the traditional dichotomy between the apogeotropic pattern and cupulolithiasis. In short-arm canalolithiasis, freely mobile particles located within the ampullary segment move away from the ampulla during the SRT when the head is turned toward the affected side, generating inhibitory ampullofugal endolymphatic flow and apogeotropic horizontal nystagmus. Conversely, contralateral head rotation produces ampullopetal flow and a stronger excitatory response, explaining why the affected ear corresponds to the side with the less intense nystagmus [6].

Clinically, this variant is characterized by apogeotropic nystagmus of short or intermediate duration (generally <60 s), with shorter latency, lower amplitude, and a more labile behavior, including frequent conversion to the geotropic form during diagnostic or therapeutic maneuvers [2,6,17]. This pattern reflects an unstable mechanical system in which small changes in the position of the otoconial debris can substantially alter the force vector acting on the cupula. As proposed by Ramos et al., careful interpretation of these nystagmus patterns may indirectly allow precise localization of the otoconial debris based on the observed sequence of eye movements, supporting the concept that apogeotropic horizontal BPPV is not a single clinical entity but rather the manifestation of distinct mechanical states within the same pathophysiological continuum [23].

### 3.3. Long-Arm Cupulolithiasis

**Figure 4:**
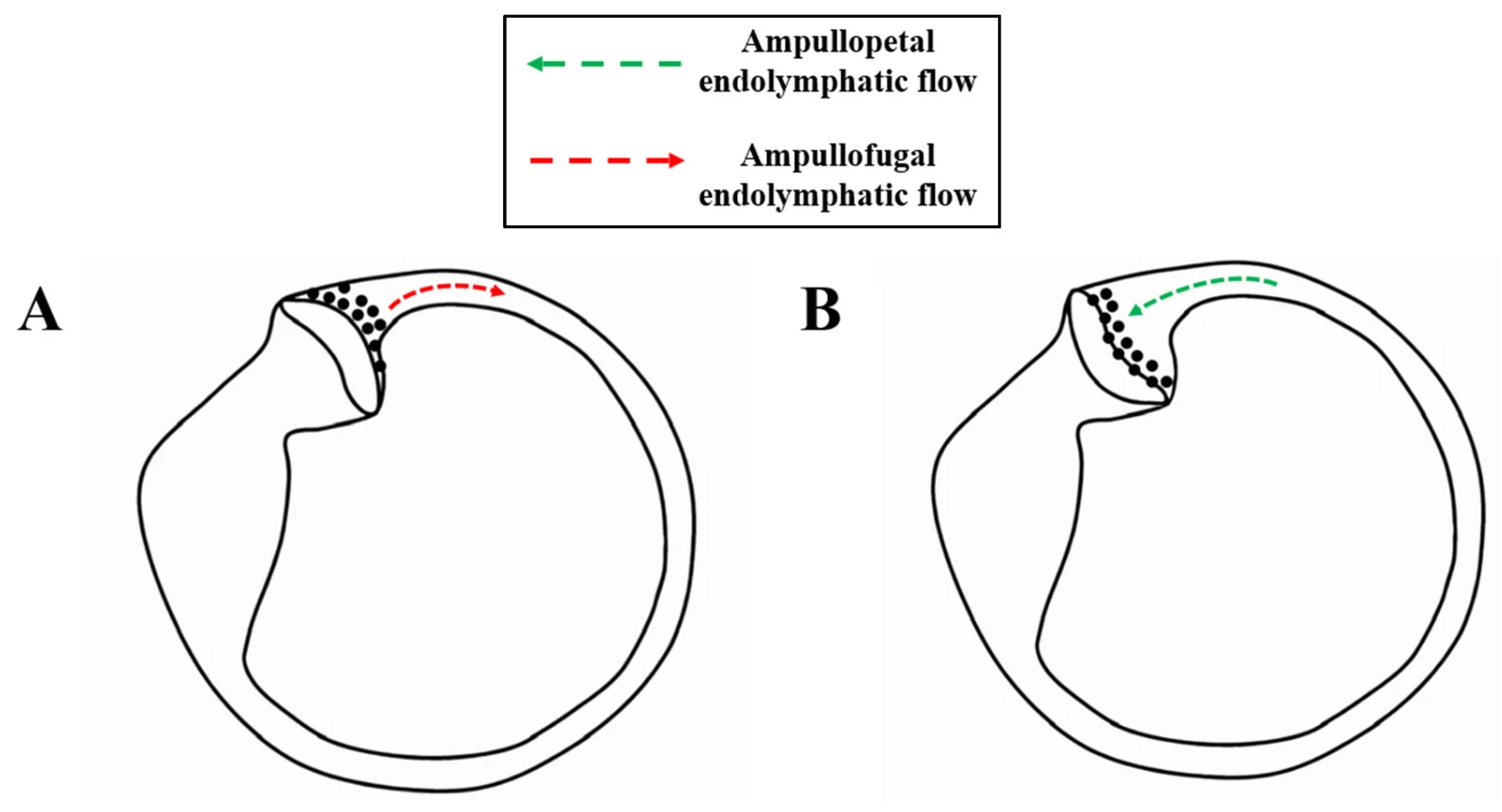
Long-arm cupulolithiasis involving the canalicular surface of the HSC cupula. (**A**) Head rotation toward the affected side: sustained predominantly ampullofugal cupular deflection, resulting in a persistent, low-intensity apogeotropic nystagmus. (**B**) Contralateral head rotation: change in head orientation relative to the cupula, producing ampullopetal cupular deflection and a more intense nystagmic response.

Long-arm cupulolithiasis, involving the canalicular surface of the HSC cupula, has traditionally been described as the attachment or impaction of an otoconial aggregate onto the canalicular aspect of the cupula (Figure 4), rendering this structure abnormally sensitive to gravity [24]. From a mechanical standpoint, this represents a qualitative change in system behavior: the cupula no longer functions as a dynamic sensor of angular acceleration but instead behaves as a structure subjected to a sustained gravitational load, whose deflection depends on head orientation relative to the gravitational vector [25]. Experimental models have shown that this condition produces a biphasic response consisting of an initial peak following otoconial impact, followed by a sustained low-amplitude deflection, considerably smaller than that observed in canalolithiasis but persistent over time [26].

This framework explains the classical clinical presentation of persistent apogeotropic variants, which are characterized by minimal latency, prolonged nystagmus lasting more than 1 min, and little or no fatigability, with an SPV that is typically lower and more stable than that observed in canalolithiasis [2]. However, the traditional interpretation of these cases as resulting from stable cupular attachment has been increasingly challenged. Recent studies suggest that many cases previously classified as cupulolithiasis may actually represent periampullary impaction or non-adherent contact with the cupula (cupular impingement), in which the otoconial debris influences cupular mechanics without permanent attachment [9]. This distinction is clinically relevant, as it may explain the coexistence of persistently apogeotropic nystagmus with higher-than-expected SPV values, suggesting the persistence of a residual dynamic component.

From a clinical perspective, we hypothesize that this mechanistic ambiguity accounts for the variable response to therapeutic maneuvers. Unlike canalolithiasis, where the objective is to redirect the otoconial debris through the canal, the therapeutic challenge in this variant lies in disrupting the particle–cupula interaction, either by detaching the debris or by converting the condition into a treatable canalolithiasis [26,27]. Furthermore, evidence indicating that a substantial proportion of apogeotropic cases may actually correspond to periampullary canalolithiasis or transitional states approaching canalith jam [28] supports the concept that canalicular cupulolithiasis should not be regarded as a rigid pathological entity but rather as one point within a pathophysiological continuum ultimately dominated by mechanisms involving mobile otoconial particles.

### 3.4. Short-Arm Cupulolithiasis

**Figure 5:**
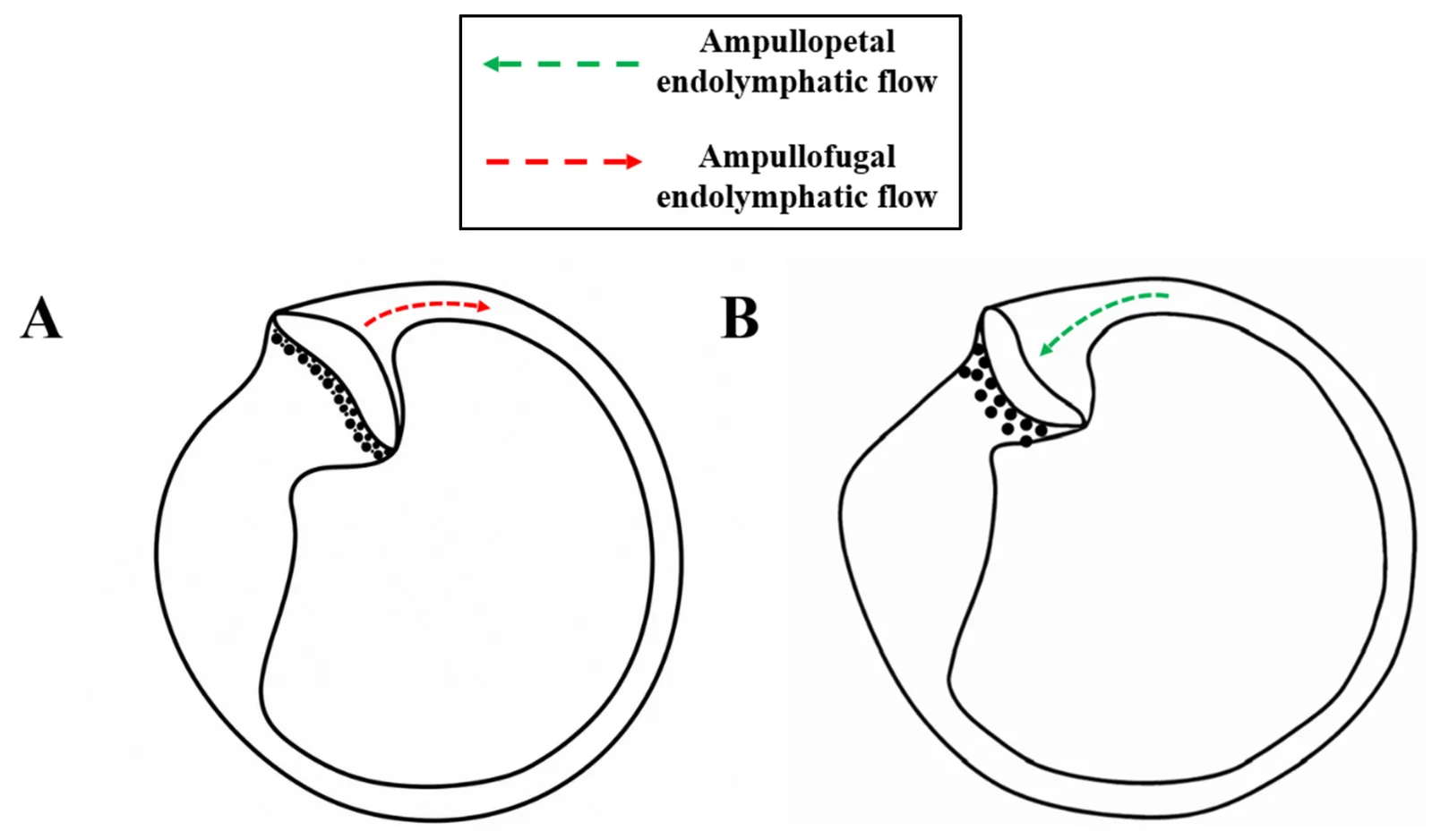
Short-arm cupulolithiasis involving the utricular surface of the HSC cupula. (**A**) Head rotation toward the affected side: sustained predominantly ampullofugal cupular deflection, generating a persistent, low-intensity apogeotropic nystagmus. (**B**) Contralateral head rotation: change in head orientation relative to the cupula, producing ampullopetal cupular deflection and a more intense nystagmic response.

Short-arm cupulolithiasis, involving the utricular surface of the HSC cupula, is a variant in which the otoconial load is attached to the vestibular aspect of the cupula (Figure 5), thereby reversing the point of application of the force vector compared with the canalicular variant [26]. This feature introduces a critical modification to the mechanical system: the applied force depends not only on the attached mass but also on the lever arm relative to the axis of cupular insertion, which in this case is oriented toward the vestibule [23]. Because the utricular surface is continuously exposed to the vestibular compartment, particle–cupula interaction may occur within a distinct hydrodynamic environment, with less confinement than on the canalicular side [29]. As a result, sustained cupular deflection develops, with both its direction and magnitude determined not only by head orientation but also by the potential redistribution of part of the otoconial load toward the utricle [30].

From a clinical perspective, both cupulolithiasis variants may produce persistent apogeotropic nystagmus (lasting >60 s) during the SRT, as well as pseudo-spontaneous nystagmus during the Bow and Lean test, as described in Section 3.1, although with a presentation opposite to that observed in long-arm canalolithiasis [31]. Nevertheless, their behavior during sequential positional maneuvers reveals important differences. When the otoconial debris is attached to the utricular surface, nystagmus tends to be more persistent, and conversion to canalolithiasis generally requires specific conditions to disrupt the particle–cupula interaction. In contrast, release from the canalicular surface may occur more readily and may even happen spontaneously [32]. Evidence derived from maneuvers such as the Zuma maneuver indicates that the differential nystagmus patterns observed across successive head positions may allow inference of the location of the otoconial debris, supporting the functional existence of the two cupular surfaces as distinct pathophysiological entities [23,27].

The concept of the null point further supports the gravitational model of cupulolithiasis. It corresponds to the head position in which the cupular axis becomes aligned with the gravitational vector, thereby eliminating cupular deflection and, consequently, nystagmus. In the HSC, this position is typically achieved with head rotation of approximately 20–30° toward the affected side, with reported mean values ranging from 24° to 27° [33]. Identification of the null point does not indicate resolution of the disorder but rather reflects a mechanically neutral configuration of the system [34].

### 3.5. Canalith Jam of the Horizontal Semicircular Canal

**Figure 6:**
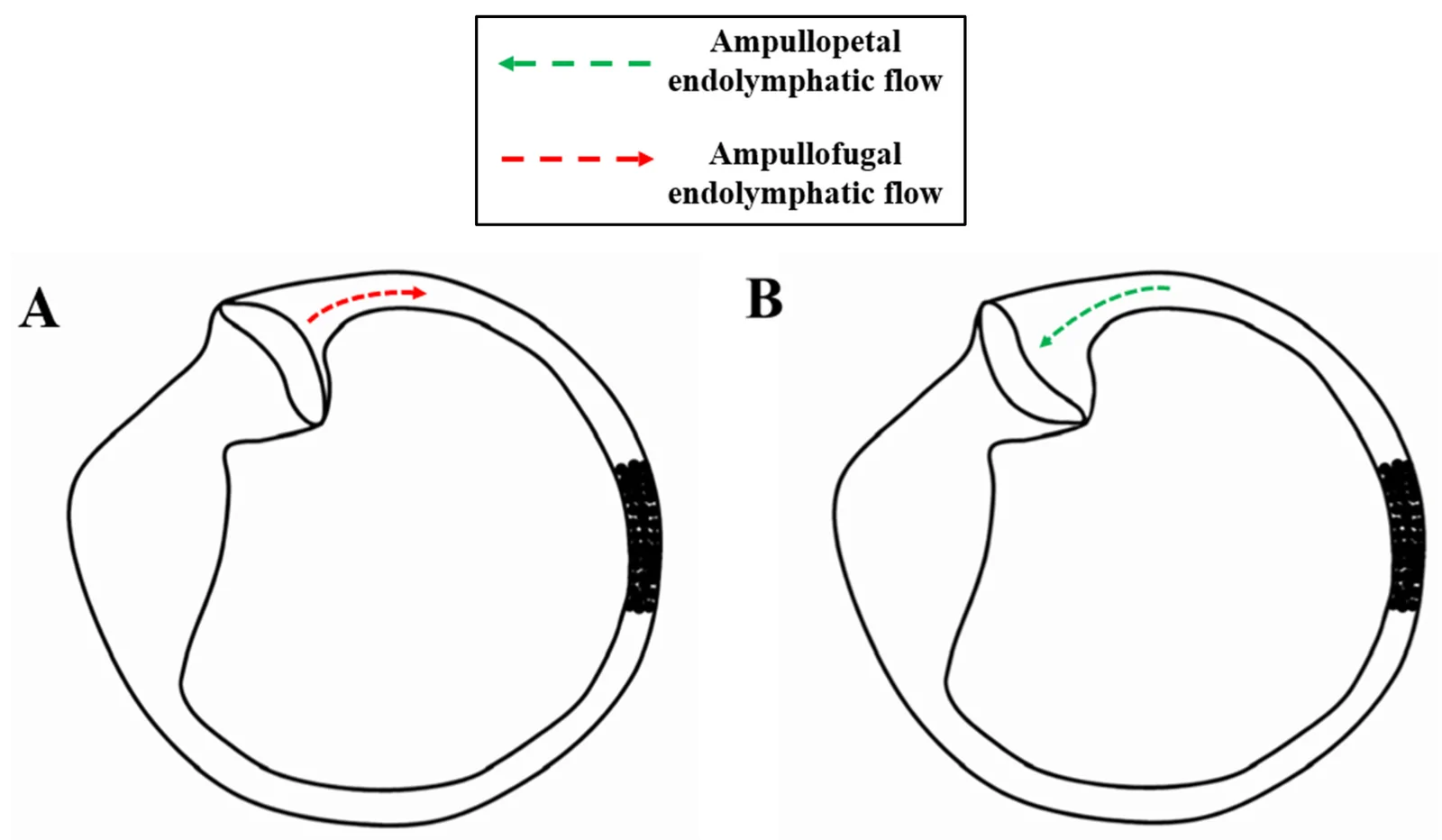
Canalith jam of the HSC. (**A**) Configuration with sustained ampullofugal cupular deflection, resulting in a fixed-direction apogeotropic horizontal nystagmus. (**B**) Configuration with sustained ampullopetal cupular deflection, generating a fixed-direction geotropic horizontal nystagmus.

Canalith jam of the HSC should be conceptualized as a state of mechanical luminal occlusion in which the otoconial aggregate no longer behaves as a freely mobile mass but instead acts as a partial or complete plug within the canal lumen (Figure 6). This hypothesis was first proposed by John Epley in 1995 [3], who described an abrupt conversion during repositioning maneuvers to an intense, persistent, position-independent nystagmus, attributing it to particle impaction within the canal. Under these conditions, the canal no longer transmits angular acceleration through endolymphatic flow but instead develops a sustained pressure gradient across the obstruction, resulting in persistent cupular deflection [7,35]. As a consequence, the direction of the resulting pressure gradient is not intrinsic to the obstruction itself but depends on the location of the impacted debris relative to the cupula and on the direction of the residual endolymphatic displacement produced by head movement. Depending on whether pressure becomes greater on the canalicular or utricular side of the cupula, the resulting deflection may be ampullopetal or ampullofugal, thereby producing geotropic or apogeotropic nystagmus. Thus, apparently opposite nystagmus patterns may arise from the same obstructive mechanism operating at different sites or under different spatial conditions.

From a clinical perspective, this configuration closely resembles vestibular neuritis, presenting with acute vertigo and spontaneous unidirectional fixed-direction nystagmus, thereby complicating its differentiation from acute vestibular syndrome (AVS) [36,37,38,39,40]. The distinguishing feature, however, lies in the functional profile. Canalith jam may be associated with isolated HSC hypofunction on the video head impulse test (vHIT), characterized by reduced vestibulo-ocular reflex (VOR) gain and corrective saccades, in the absence of otolithic dysfunction or involvement of the remaining semicircular canals. This pattern of “pseudo-hypofunction” resolves following release of the obstruction, confirming its mechanical rather than structural origin [35]. Consistent with this mechanism, ipsilateral canal paresis with complete functional recovery after repositioning maneuvers has also been reported [7].

Recent studies have demonstrated that canalith jam is not necessarily a static condition. Reversible forms have been described in which the otoconial aggregate alternates between becoming impacted at narrow segments of the canal and contacting the cupula, even functioning as a “false cupula” that modulates nystagmus velocity without altering its direction [7,41]. This dynamic behavior explains phenomena such as geotropization, in which a contralesional fixed-direction nystagmus converts to a geotropic pattern following release of the obstruction. From this perspective, canalith jam should not be regarded as a distinct pathological entity but rather as the most extreme manifestation within the spectrum of canalolithiasis, in which the otoconial aggregate reaches sufficient size and configuration to obstruct the canal lumen and qualitatively alter ampullary mechanics.

### 3.6. Light Cupula

**Figure 7:**
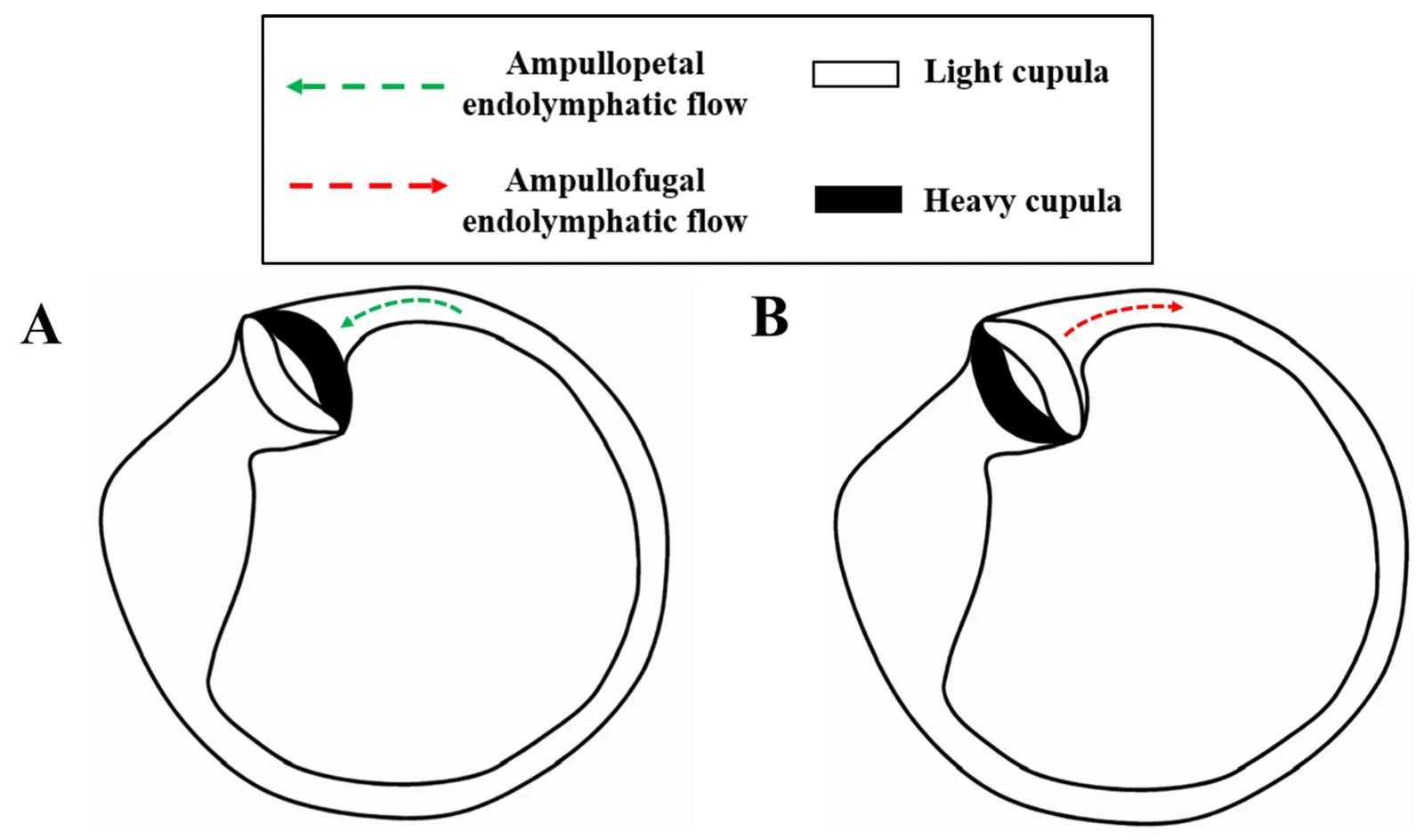
Light cupula of the HSC. Schematic representation of the gravity-dependent behavior of light (white) and heavy (black) cupula. (**A**) Head rotation toward one side: the density difference induces sustained cupular deflection, resulting in persistent geotropic nystagmus. (**B**) Contralateral head rotation: a change in orientation relative to the gravitational vector reverses the direction of cupular deflection while maintaining persistent geotropic nystagmus in the opposite direction.

Light cupula cannot be incorporated into the classical otoconial models because its underlying mechanism is not based on the presence of otoconial debris but rather on an alteration in the relative densities of the cupula and the surrounding endolymph. Under normal conditions, these two structures are isodense, rendering the system insensitive to gravity. When the cupula becomes relatively less dense than the surrounding endolymph, buoyancy forces emerge, subjecting the cupula to a sustained upward force that produces continuous deflection dependent on head orientation, without the need for transient endolymphatic flow [40]. This model directly explains the occurrence of persistent geotropic nystagmus (Figure 7), characterized by the absence of latency and fatigability, together with prolonged time constants (>60 s) typical of systems dominated by static forces [41]. Conversely, the heavy cupula model represents the opposite end of the same pathophysiological continuum: a cupula denser than the surrounding endolymph produces a persistent nystagmus pattern with a symmetrical but inverted relationship between head position and slow-phase velocity (SPV) compared with light cupula, supporting the concept that both conditions share the same underlying mechanism but with opposite directions of cupular deflection [42].

In this setting, SPV varies continuously with the angle of head rotation, following an approximately linear relationship around a neutral plane (the null point), which is typically displaced 15–30° toward the affected side [43]. On either side of this neutral plane, the direction of nystagmus reverses symmetrically, resulting in an almost linear relationship between head rotation angle and SPV, further supporting the gravitational nature of the phenomenon [41]. Conceptually, the light cupula model requires abandoning the paradigm of transient pressure changes generated by freely mobile particles in favor of a model of sustained cupular deflection driven by density differences, resembling the mechanisms described in positional alcohol nystagmus more closely than those underlying conventional BPPV [40].

From a therapeutic perspective, it is important to emphasize that light cupula is characterized by a poor or inconsistent response to canalith repositioning maneuvers, representing a key distinguishing feature from the other HSC-BPPV variants. Because there is no mobile otoconial substrate to reposition, maneuvers designed to mobilize otoconial debris are frequently ineffective or produce unpredictable responses, further supporting a mechanism based on density differences rather than displaced otoconial material [41,44,45,46]. Consequently, management is often more challenging, with symptoms typically persisting for days to weeks before resolving spontaneously in many cases.

**Table 1:** Summary of otoneurological findings and therapeutic options according to the HSC-BPPV variant. FPP: Forced Prolonged Position; CuRM: Cupulolithiasis Repositioning Maneuver.

HSC Positional Vertigo Configuration	Cupular Deflection		Nystagmus Direction		Nystagmus Duration	Reposition Maneuvers [23,24,30,32]
	Healthy SRT	Affected SRT	Healthy SRT	Affected SRT		
**Long-arm** **canalolithiasis**	Ampullofugal	Ampullopetal	Weaker geotropic	Stronger geotropic	<60 s.	Lempert Gufoni Asprella Vannucchi-Asprella Li maneuver FPP
**Short arm** **canalolithiasis**	Ampullopetal	Ampullofugal	Stronger apogeotropic	Weaker apogeotropic	<60 s.	
**Long-arm** **cupulolithiasis**	Ampullopetal	Ampullofugal	Stronger apogeotropic	Weaker apogeotropic	>60 s.	Zuma e Maia Gufoni-Appiani Gufoni + Lempert Modified Semont CuRM
**Short arm** **cupulolithiasis**	Ampullopetal	Ampullofugal	Stronger apogeotropic	Weaker apogeotropic	>60 s.	
**Canalith jam**	Ampullopetal or Ampullofugal		Fixed-direction geotropic/apogeotropic		>60 s.	Lempert Gufoni
**Light cupula**	Variable depending on density		Geotropic	Geotropic	>60 s.	-

## 4. Discussion

The available evidence suggests that HSC-BPPV variants should no longer be interpreted exclusively through the classical dichotomy of “geotropic = canalolithiasis” and “apogeotropic = cupulolithiasis.” Instead, the contemporary literature increasingly supports a continuum model in which the observed nystagmus pattern depends primarily on the location of the otoconial debris, its mechanical state, and its dynamic interaction with the cupula, rather than on mutually exclusive pathophysiological mechanisms [2,21,22].

For otoconial disorders, otoconial location appears to represent the principal organizing factor underlying HSC pathophysiology. Long-arm canalolithiasis behaves predominantly as a hydrodynamic phenomenon, in which gravity-induced endolymphatic flow produces transient cupular deflection, typically resulting in geotropic nystagmus characterized by high slow-phase velocity (SPV), fatigability, and a duration of less than 60 s [10]. The requirement to flex the head approximately 30° while the patient is in the supine position to align the HSC plane with the gravitational vector is not merely a technical detail but rather a fundamental physical prerequisite for maximizing particle displacement and endolymphatic flow generation [13,16]. This explains both the high reproducibility of the supine roll test (SRT) and the consistent SPV asymmetry used to identify the affected side [17].

Conversely, recent studies suggest that the apogeotropic pattern does not necessarily imply true adhesion of otoconial debris to the cupula. Using the Zuma maneuver as a dynamic diagnostic tool, Ramos et al. demonstrated that different mechanical configurations—including periampullary canalolithiasis, canalicular cupulolithiasis, and utricular cupulolithiasis—may all generate similar apogeotropic responses during the SRT [23,27,32]. These findings challenge the traditional association between nystagmus direction and a specific pathophysiological mechanism. Rather, nystagmus direction likely represents only the final manifestation of a complex mechanical system determined by the location of the otoconial debris, the spatial orientation of the canal, and the dynamic interaction between the particles and the cupula.

Accordingly, one of the most important recent conceptual developments has been the critical reassessment of cupulolithiasis itself [2,4]. Stable adhesion of otoconia to the cupula remains largely a theoretical hypothesis, with little direct histopathological confirmation. Foster and Kalmanson [26] critically reviewed this model and concluded that more than 90% of BPPV cases are more plausibly explained by freely mobile particles than by persistent cupular attachment. Along similar lines, Epley proposed alternative mechanisms such as cupular impingement, in which otoconial debris dynamically contacts the cupula without permanent attachment [3]. This model provides a more convincing explanation for several clinical phenomena that are difficult to reconcile with a fixed static load, including spontaneous conversion between variants, variability in SPV, and partial responses to specific repositioning maneuvers [36].

Particularly illustrative are the three-dimensional recordings published by Imai et al., in which the transition from persistent apogeotropic patterns to geotropic variants was accompanied by a progressive increase in SPV and a marked shortening of the time constant [17]. These findings support the concept that many persistent contralesional nystagmus patterns do not represent fixed anatomical states but rather dynamic mechanical configurations capable of evolving into true canalolithiasis. The observation of time constants exceeding 100 s during cupular phases, followed by abrupt reductions after conversion to geotropic forms, constitutes one of the strongest physiological arguments supporting this interpretation.

Canalith jam probably represents the most extreme manifestation of this pathophysiological continuum. In these cases, the system ceases to function as a freely flowing endolymphatic system and instead behaves as a partially obstructed canal capable of generating sustained pressure gradients [35]. Clinically, this results in fixed-direction horizontal nystagmus and vestibular neuritis-like presentations that may occasionally be associated with genuine isolated HSC hypofunction on the video head impulse test (vHIT) [37,38,39,40]. Particularly noteworthy are the cases reported by Castellucci et al., in which patients initially diagnosed with acute vestibular syndrome were ultimately found to have canalith jam [35]. These observations are clinically relevant because they demonstrate that, in patients presenting with convincing vestibular neuritis mimics, compatible functional testing, and an appropriate clinical examination, a repositioning maneuver may completely resolve both the symptoms and the abnormal findings on vestibular testing [7].

Similarly, gravity-dependent disorders, particularly light cupula, represent an even more profound conceptual shift by moving the focus from particle motion to cupular mechanics. In these conditions, the underlying abnormality is not necessarily the presence of mobile otoconial debris but rather a density mismatch between the cupula and the surrounding endolymph, transforming a physiologically gravity-neutral structure into one that is gravity sensitive [41,43]. Imai et al. demonstrated that patients with persistent geotropic nystagmus exhibit an almost linear relationship between head angle and SPV around the null point, together with time constants exceeding 35 s and patterns that are symmetrical to those observed in heavy cupula [44]. Such behavior cannot be adequately explained by conventional canalolithiasis models and instead supports the existence of systems dominated by static gravitational forces rather than transient endolymphatic pressure gradients.

Another particularly interesting observation is the marked clinical heterogeneity of persistent nystagmus. Si et al. demonstrated that persistent geotropic forms respond less favorably to repositioning maneuvers and are more frequently associated with migraine and autoimmune disorders, whereas persistent apogeotropic forms appear to be more closely related to vascular mechanisms and, in some cases, central nervous system disorders [33]. This concept is further reinforced by recent clinical evidence showing that atypical forms of BPPV are encountered more frequently than previously recognized in specialized vestibular centers. These variants often exhibit transitional features and more complex therapeutic behavior, highlighting that positional vertigo cannot always be adequately explained by the traditional dichotomous classification and instead reflects a broader spectrum of mechanical conditions [47,48].

Finally, as described by Büki et al., the existence of subjective BPPV without observable nystagmus further supports the concept of a pathophysiological continuum. The occurrence of typical BPPV symptoms in the absence of detectable nystagmus suggests that subtle differences in the magnitude of the mechanical stimulus or in vestibular sensitivity may profoundly influence the clinical expression of the disorder [21]. From this perspective, the different HSC-BPPV variants should not be regarded as rigid and independent entities but rather as dynamic mechanical configurations capable of evolving from one form to another.

To conclude, these findings should be interpreted in light of several limitations. Because the proposed mechanical configurations cannot be directly visualized in vivo, they are largely inferred from nystagmus characteristics, responses to positional maneuvers, and biomechanical models. Moreover, the available evidence is heterogeneous and predominantly observational. The proposed continuum should therefore be considered a unifying pathophysiological model that requires prospective validation rather than a series of anatomically confirmed states. Middle-ear conditions should also be considered when interpreting vestibular findings, as marked pressure abnormalities or Eustachian tube dysfunction may affect pressure-sensitive symptoms or tests based on acoustic or pressure transmission. However, their relevance to the gravity-dependent positional nystagmus of HSC-BPPV remains uncertain, and it represents a relevant area for future interdisciplinary investigation.

## 5. Conclusions

HSC-BPPV may be more appropriately understood as a spectrum of mechanical states rather than as a collection of completely distinct entities. The underlying configuration of the otoconial debris and its interaction with the cupula appear to largely determine the resulting nystagmus pattern, clinical presentation, and subsequent evolution of the disorder. Contemporary evidence challenges the traditional view that cupulolithiasis invariably represents permanent adhesion of otoconia to the cupula and instead supports a more dynamic and integrative pathophysiological model. A deeper understanding of these mechanisms may improve diagnostic accuracy and guide the selection of therapeutic maneuvers for each clinical variant. Future studies should prospectively validate the proposed continuum using standardized positional testing and quantitative nystagmus analysis, supported by computational biomechanical modeling and, when technically feasible, advanced imaging methods capable of providing more direct evidence of particle location and cupular behavior.

## Data Availability

No new data were generated or analyzed in this study. All information supporting the findings of this review is available in the published literature cited in the reference list.
